# Metformin in therapeutic applications in human diseases: its mechanism of action and clinical study

**DOI:** 10.1186/s43556-022-00108-w

**Published:** 2022-12-09

**Authors:** Yang Du, Ya-Juan Zhu, Yi-Xin Zhou, Jing Ding, Ji-Yan Liu

**Affiliations:** 1 Department of Biotherapy, Cancer Center, State Key Laboratory of Biotherapy, West China Hospital, West China Medical School, Sichuan University, Chengdu, China; 2 Department of Medical Oncology, Sichuan Cancer Hospital & Institute, Sichuan Cancer Center, School of Medicine, University of Electronic Science and Technology of China, Chengdu, Sichuan China

**Keywords:** Metformin, AMPK, Redox balance, Mitochondria, Gut microbiome, Adverse effects

## Abstract

Metformin, a biguanide drug, is the most commonly used first-line medication for type 2 diabetes mellites due to its outstanding glucose-lowering ability. After oral administration of 1 g, metformin peaked plasma concentration of approximately 20–30 μM in 3 h, and then it mainly accumulated in the gastrointestinal tract, liver and kidney. Substantial studies have indicated that metformin exerts its beneficial or deleterious effect by multiple mechanisms, apart from AMPK-dependent mechanism, also including several AMPK-independent mechanisms, such as restoring of redox balance, affecting mitochondrial function, modulating gut microbiome and regulating several other signals, such as FBP1, PP2A, FGF21, SIRT1 and mTOR. On the basis of these multiple mechanisms, researchers tried to repurpose this old drug and further explored the possible indications and adverse effects of metformin. Through investigating with clinical studies, researchers concluded that in addition to decreasing cardiovascular events and anti-obesity, metformin is also beneficial for neurodegenerative disease, polycystic ovary syndrome, aging, cancer and COVID-19, however, it also induces some adverse effects, such as gastrointestinal complaints, lactic acidosis, vitamin B12 deficiency, neurodegenerative disease and offspring impairment. Of note, the dose of metformin used in most studies is much higher than its clinically relevant dose, which may cast doubt on the actual effects of metformin on these disease in the clinic. This review summarizes these research developments on the mechanism of action and clinical evidence of metformin and discusses its therapeutic potential and clinical safety.

## Introduction

**Fig. 1:**
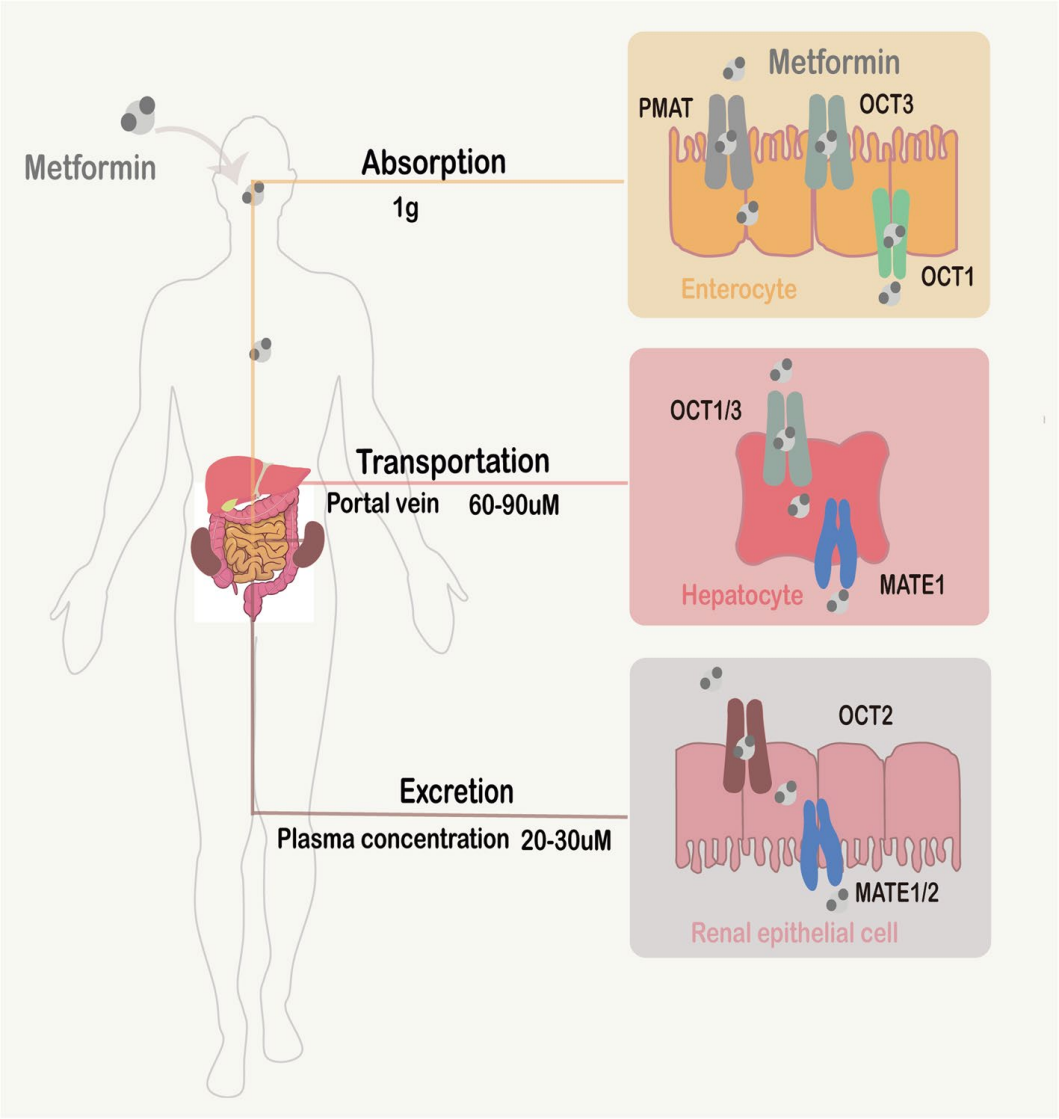
Pharmacokinetics of Metformin. Following oral dosing of 1 g metformin, the uptake of metformin in intestinal, liver and kidney is mediated by PMAT/OCT3, OCT1/3 and OCT2, respectively, and the excretion of metformin in intestinal, liver and kidney is mediated by OCT1, MATE1 and MATE1/2, respectively. The plasma concentrations of metformin are between 20–30 μM, and the concentrations of metformin in the portal vein are 60–90 μM

Metformin, derivated from biguanide, is able to effectively lower plasma glucose level by inhibiting hepatic gluconeogenesis (HGP) and improving insulin-resistance with high cost-effectiveness, but nearly has no hypoglycemia side effects [1, 2]. Therefore, since it was synthesized in the 1920s, metformin has been the recommended first-line medication for type 2 diabetes mellites (T2DM) [3]. Metformin is not metabolized and is eliminated unchanged through renal excretion, and this drug is widely distributed into various organs, including intestinal, liver, kidney, brain and so on. After oral administration, metformin is first absorbed in the intestine, which is mediated by plasma membrane monoamine transporter (PMAT) or organic cation transporter 3 (OCT3) on the luminal side of enterocytes [4, 5]. Then, metformin leaves the enterocytes and is transferred into the portal vein through OCT1 on the basolateral membrane. Next, metformin is delivered to the liver and absorbed via OCT1/OCT3, which is expressed on the basolateral membrane of hepatocytes [6, 7], and is excreted from the liver to the circulation via multidrug and toxin extrusion 1(MATE1) [8]. Last, metformin in the circulation is absorbed into renal epithelial cells, which is mediated by OCT2 on the basolateral membrane in the renal tubules [9], and further eliminated into urine through MATE1 and MATE2-K on the apical membrane of the renal proximal tubule cells [10, 11] (Fig. 1).

Accordingly, following oral dosing of 1 g metformin, a prescribed dose for T2DM treatment in the clinic, the plasma concentrations of metformin are between 20–30 μM, and the concentrations of metformin in the portal vein are roughly estimated to be threefold higher. Therefore, following a therapeutic dose, the hepatic exposure to metformin ranges from 60–90 μM [12]. To explore the clinically relevant doses of metformin in preclinical studies, Madiraju et al. compared the hepatic exposures in rats following different oral ingestions of metformin, and he found that the hepatic exposure to metformin (approximately 50–100 μM) is similar between oral ingestions of 50–100 mg/kg metformin in rats and oral ingestions of 1 g metformin in humans. And the oral dosing of ≥ 250 mg/kg metformin results in > 1 mM hepatic exposure to metformin [13, 14].

Recently, researchers have further explored the underlying mechanisms of action mediated by metformin. One of the most studies mechanisms is the activation of AMP-activated protein kinase (AMPK) [15, 16], a key regulator of various pathways involved in glucose, lipid and energy metabolism. For example, the blockade of AMPK signaling significantly influences the efficiency of metformin for T2DM and atherosclerosis [17, 18]. Besides, metformin also plays roles in changing the pathogenesis of diseases by restoring redox balance, affecting mitochondrial function, activating protein phosphatase 2 (PP2A), releasing fibroblast growth factor 21(FGF21) and so on [19–23]. Moreover, metformin even enables the modulation of gut microbiota [24, 25].

Due to the board mechanisms of action, despite of T2DM, new applications of this old drug have been investigated, such as decreasing cardiovascular events and anti-obesity [26–29]. In addition, evidence is accumulating that metformin also has potential benefits for cancer [30–32], neurodegenerative disease [33, 34], metabolic syndrome [35, 36], polycystic ovary syndrome (PCOS) [37–39], aging [40–42], coronavirus disease 2019 (COVID-19) [43–45]and so on. However, metformin also results in some adverse effects, such as gastrointestinal complaints, lactic acidosis, vitamin B12 deficiency and neurodegenerative disease [46–48]. Recently, it has even been reported that metformin treatment during the sperm development increased the risk of birth defects in offspring [49].

However, compared to clinical concentration for treating T2DM patients, much higher metformin concentrations are widely used in beforementioned studies. Hence, although multiple pharmacological effects and clinical evidences have been reported, the mechanisms of action and new applications of this most commonly antidiabetic drug remains only partially elucidated and controversial, especially the metformin dosage in researches, its clinical use is now still limited to diabetic patients. Here, we will summarize and analyze recent research developments on the mechanism of action and clinical evidence of metformin, helping to better understand and repurpose metformin.

## Mechanism of action

Metformin is reported to have a number of targets, the first and studied most is AMPK-signaling. Then, researchers found metformin can still affect cells in the absence of AMPK through targeting redox states, mitochondria, and some other signaling, such as FGF21, PP2A, and mTOR. Furthermore, metformin also modulates the gut microbiome to indirectly regulate the human homeostasis. In this part, we will first discuss these mechanisms of action mediated by metformin.

### Metfromin exerts its effect in an AMPK-dependent manner

**Fig. 2:**
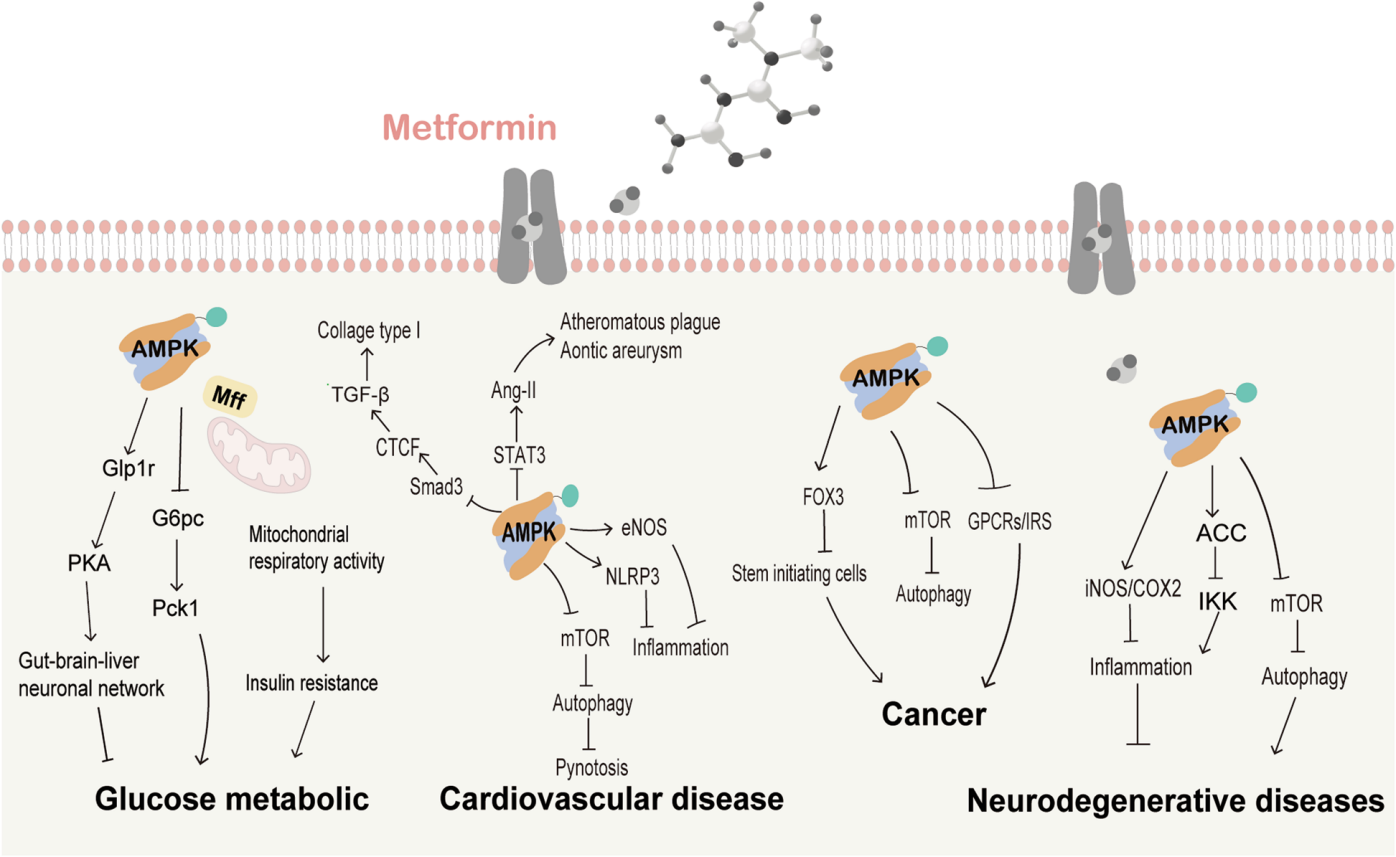
AMPK-dependent mechanism of action mediated by metformin treatment. Depending on AMPK activation, metformin exerts its effects by regulating Glp1r/Pka pathway, mTOR/autophagy pathway, NLRP3, eNOS, STAT3,COX-2, iNOS, Smad3, FOX3, IRS, GCRP and PD-L1 and ACE2 signals

Numerous literatures have demonstrated that metformin exerts its effect through AMPK activation (Fig. 2). AMPK is a heterotrimeric complex, consisting of the α catalytic subunit, scaffold protein β subunit and regulatory γ noncatalytic subunit [50]. The activation of AMPK is initiated by the binding of adenosine monophosphate (AMP) to the γ-subunit, which can lead to structural changes in AMPK and then induce the phosphorylation of the α subunit at Thr^172^. Based on this mechanism, metformin may mediate AMPK activation by increasing the AMP/ATP (adenosine triphosphate). Interestingly, it has been reported that metformin could also directly bind to the γ subunit of AMPK, however, it is still unclear that whether this interaction between metformin and the γ-subunit can directly activate AMPK, such as AMP [51]. In addition, following glucose starvation, low-dose metformin (5–30 μM) also could activate AMPK through binding with the presenilin enhancer (PEN2) to inhibit the lysosomal proton v-ATPase, while the phosphorylation of AMPK could be suppressed by imidazole propionate, a microbial metabolite, via activating the p38g/AKT (also known as protein kinase B or PKB) pathway [52, 53].

Because of its role in the reduction of acetyl-CoA carboxylase (ACC) activity and lipogenic enzymes and the induction of fatty acid oxidation, AMPK is reported to be as a key regulator in lipid and glucose metabolism [54]. Furthermore, AMPK is also involved in a number of pathways, such as mammalian target of rapamycin complex1(mTORC1) signaling, peroxisome proliferator-activated receptor γ coactivator 1 (PGC-1) signaling and signal transducer and activator of transcription 3 (STAT3) signaling [55–57]. Consequently, the various effects of AMPK may be partly responsible for metformin’s the wide effects on homeostasis and diseases. As a classical antidiabetic drug, metformin was discovered to lower plasma glucose levels by reducing hepatic glucose production (HGP) and alleviating insulin resistance. Cao et al. reported that 80 μM metformin, a therapeutic metformin concentration in the portal vein, is enough to decrease glucose production and the mRNA levels of glucose-6-phosphatase catalytic (*G6pc)* and phosphoenolpyruvate carboxykinase 1 (*Pck1)* in primary hepatocytes in an AMPK-dependent way [58]. Furthermore, Frank et al. demonstrated that a clinically relevant dose of metformin treatment (50 mg/kg) in rats could initiate the AMPK- glucagon like peptide 1 receptor (Glp1r)- protein kinase A (PKA) pathway in the duodenal mucosa, and then enhance the HGP inhibitory effect of metformin depending on the gut-brain-liver neuronal network [59, 60]. Besides, pharmacological metformin concentration (75 μM) treatment of hepatocytes was reported to improve mitochondrial respiratory activity and increase ATP levels by increasing mitochondrial oxidative enzymes and promoting mitochondrial fission through AMPK/mitochondrial fission factor (Mff) signaling. As it has been widely accepted that impaired mitochondrial respiratory activity is a key inducer for the development of insulin resistance, it is reasonable that a pharmacological dose of metformin can improve insulin resistance by activating AMPK [61].

Apart from benefits for T2DM, metformin is also able to improve cardiovascular diseases through reducing cardiovascular end points, not just because of its glucose-lowering effect. The role of AMPK in this metformin-mediated cardiovascular protective action has been elucidated in number of literatures. After treating streptozotocin-induced diabetic cardiomyopathy (DCM) mice with 200 mg/kg metformin and high glucose-treated cardiomyocytes with 2 mM metformin, Fan et al. found that metformin improves autophagy and then alleviates the pyroptosis in DCM by inhibiting the AMPK/mTOR pathway [62]. This AMPK/mTOR-mediated inhibition of autophagy also drives neuroprotection against focal cerebral ischemia after acute preconditioning with a subtherapeutic dose of metformin (10 mg/kg, i.p.) [63]. Moreover, by activating the AMPK/mTOR pathway, metformin is a potential therapeutic for other neurological diseases, such as Parkinson’s disease (PD) and Huntington’s disease, through enhancing neuronal bioenergetics, protecting nerve repair and reducing toxin protein aggregates [64].

Moreover, metformin is also reported to be beneficial for patients with inflammatory diseases. Metformin inhibits the inflammatory response through activating the AMPK/ NLR family pyrin domain containing 3(NLRP3) or AMPK/ endothelial nitric oxide synthase (eNOS) pathway, thus protecting the myocardial from ischemia–reperfusion [65, 66]. In addition, the antiatherosclerosis role of metformin has also been documented, relying on AMPK activation. A therapeutic dose of metformin (100 mg/kg) inhibits monocyte-to-macrophage differentiation and proinflammatory cytokine production via sequentially decreasing STAT3 phosphorylation and attenuating Angiotensin (Ang)-II-induced atheromatous plaque formation and aortic aneurysm in an atherosclerosis mice model [17]. Researches have also pointed out that T2DM-linked neurodegenerative disease (ND), such as Alzheimer’s disease (AD), is related to advanced glycosylation end product (AGE)-caused neuronal impairment via the inflammatory response, and metformin (1 mM) could rescue this inflammation-induced impairment through upregulating of ACC and inhibitory kappa B kinase (IKK), accompanied by restoring inducible nitric oxide synthase (iNOS) and cyclooxygenase-2(COX-2) in an AMPK-dependent way [67]. As metformin plays its cardiovascular protective and neuroprotective roles by exerting an inflammatory inhibitory effect, researchers have further investigated the role of metformin in inflammatory diseases. By utilizing a partial medical meniscectomy (DMM) model, Chen et al. found that metformin (205 mg/kg) inhibits cartilage degradation and limits osteoarthritis development and progression in an AMPK-dependent way [68]. Besides, it has also been illustrated that with the activation of AMPK, metformin (10 mM) reduces transforming growth factor beta (TGF-β)-induced renal fibroblast collagen type I production via inhibiting Smad3-driven connective tissue growth factor (CTGF) expression, and this mechanism may provide a potential role for metformin in the treatment of chronic kidney disease (CKD) through suppressing renal interstitial fibrosis [69].

Accordingly, the AMPK-dependent effect of metformin has been implied to be beneficial for many other pathogeneses, such as cancer [70]. For the critical role of cancer stem/initiating cells in tumorigenesis and cancer development, researchers explored whether metformin affects cancer initiating cells, and found that metformin (1 mM) activates hexaribonucleotide-binding protein 3(FOX3) via AMPK activation, which is sufficient to promote the differentiation of glioma-initiating cells into nontumorigenic cells [71]. Moreover, growing evidences have indicated that metformin has direct therapeutic potential for cancers, whether as a sole drug or in combination with other regimens. AMPK activation by metformin (up to10 mM for cells and 300 mg/kg for mice models) induces autophagy through inhibiting mTOR signaling or the immune response, and thus downregulates programmed death-ligand 1 (PD-L1) expression in a variety of cancer types, such as lymphoma, breast cancer, pancreatic cancer, non-small cell lung cancer, eventually, the growth or metastasis of cancer cells is inhibited [72–76]. It has also been demonstrated that following activation of AMPK, metformin (5 mM for cells and 250 mg/kg for mice models) inhibits pancreatic cancer growth by disrupting the insulin receptor signaling (IRS) or G protein coupled receptor systems (GPCRs) [77–79]. Moreover, metformin (100 μM-10 mM for cells and 300–500 mg/kg for mice models) also amplifies its therapeutic effects and enhances cancer patient survival beneficial in an AMPK-dependent way when combined with radiotherapy or chemotherapy [80, 81].

As AMPK is widely expressed in the ovary and testes, so the role of metformin, an AMPK activator, in the reproductive system has also attracted much interest from researchers. The results showed that through activating AMPK-cyclic AMP (cAMP) signaling, metformin (10 mM) has a positive effect on polycystic ovary syndrome (PCOS), a disease associated with reproductive and metabolic abnormalities, by inhibiting steroidogenic enzymes and decreasing androstenedione production [82–85]. To add, it is well documented that the inhibition of testicular AMPK is an important contributor to the impairment of spermatogenesis and steroidogenesis, so there is reason to believe that in the patients with T2DM or other metabolic disorders, metformin’s restorative role in male reproductive dysfunction is mainly through normalizing of AMPK in testes [86–89].

Coronavirus disease 2019 (COVID-19), a currently leading threat to public health and the economy, is caused by severe acute respiratory syndrome coronavirus-2 (SARS-CoV-2). The SARS-CoV-2 has strong binding affinity to the angiotensin covering enzyme 2(ACE2) receptor of pneumocystis and enterocytes, which is essential for virus entry into cells and leads to its rapid spread throughout the world [90]. On the other hand, ACE2 signaling protects against from COVID-19 complications by regulating the renin–angiotensin–aldosterone system (RAAS) to exert antihypertensive and anti-inflammatory effects [91–93]. Zhang et al. demonstrated that the activation of AMPK could cause the Ser-680 phosphorylation of ACE2, thus resulting in inhibiting of the binding of ACE2-viral spike protein, extending the half-life and increasing stability of ACE2 [94, 95]. Taken together, the possible molecular basis for the beneficial role of metformin in COVID-19 complications is also associated with metformin-mediated AMPK activation. However, the underlying mechanisms and theoretical potential for metformin as a treatment for COVID-19 need to be further investigated and confirmed.

However, AMPK activation may also lead adverse effects on homeostasis or disease. Researches have suggested that AMPK signaling, simulated by maternal hyperglycemia-induced embryo oxidative stress, could disrupt embryonic gene expression, so it may cause neural tube defects. To confirm this hypothesis, Loeken and his colleagues investigated whether AMPK activator metformin has a similar adverse effect on embryos, however, the results indicated that due to the lack of the metformin transporters, MATE1 and OCT3, metformin (120 mg/kg) has no influence on AMPK activation in embryos, and did not cause consequent neural tube defects during pregnancy [96]. In Other researches, the evidence implied that an over-dose (2 mM) of metformin indeed has adverse effects; for example, AMPK activation inhibits MIN6 pancreatic β cells proliferation and promotes apoptosis in vitro, which is the underlying mechanism of metformin-induced acute pancreatitis in patients with renal insufficiency [97]. Overactivation of AMPK by metformin (100 μM -1 mM) also inhibits axon growth, impairs neuronal polarization, and even dendritic spine loss, which is related to the early stage of AD [98, 99] (Table 1).

**Table 1:** Dosages of metformin in In vitro and In vivo experiments

Mechanisms	Model	Dose	Route of Administration	Duration/Frequency	Reference
AMPK signaling	Mice	50 mg/kg	Oral	6 weeks	[58]
	Cells	80 μM		4 weeks	
	Rats	50 mg/kg			[59]
	Cells	75 μM			[61]
	Mice	200 mg/kg	Oral	8 weeks	[62]
	Cardiomyocytes	2 mM		24 h	
	Rats	10 mg/kg	i.p		[63]
	Mice	100 mg/kg	Oral	6 weeks	[17]
	THP-1 cells	2 mM		24 h	
	hNSCs	1 mM			[67]
	Rats	205 mg/kg	Oral	6 and 12 weeks	[68]
	Fibroblasts	10 mM			[69]
	Cells	1 mM			[71]
	Mice	300 mg/kg	Oral	2 weeks	[72]
	Cells	10 mM		24 h	
	Mice	250 mg/kg	i.p	24–36 days	[78]
	Cells	5 mM			
	Mice	300–500 mg/kg	Oral	21 days	[80, 81]
	Cells	0.1–10 mM			
	Cells	10 mM			[85]
	Cells	2 mM			[97]
	Neurons	0.1–1 mM			[98, 99]
Redox state	Rats	50 mg/kg	i.v		[14]
	Cells	1–10 mM			[100–104]
	Rats	300 mg/kg	Oral	30 days/4 weeks	[105, 106]
	Cells	10 mM			[107]
	T cells	0.1 mM			[42]
	Mice	150 mg/kg	Oral	4 days	[108]
	Cells	1 mM			
Mitochondria	Cells	≥ 250 μM			[109]
	Mice	500 mg/kg	Oral		[110]
	Hepatocytes	125 μM			
	Mice	250 mg/kg	Oral	10 weeks	[111]
	Mice	500 mg/kg	Oral	2 weeks	[20, 112]
	Cells	1 mM			
	Cells	500 μM			[113]
	Cells	0.05–2 mM			[114, 115]
	Worm	50 μM			[116]
	Fibroblasts	500 μM			[117]
Gut microbiome	Mice	200 mg/kg	Oral	1 week	[24, 118]
	Mice	100 mg/kg	Oral	11 weeks	[119]
	Mice	250 mg/kg	Oral	16 weeks	[25]
	Mice	250 mg/kg	Oral	2 months	[120]
	Cells	50 mM			[121]
FBP1	Mice	250 mg/kg	Oral	2 h	[122]
PP2A	Cells	0.5 mM			[123]
	Mice	200 mg/kg	Oral	4 weeks	[124]
FGF21	Mice	10/50 mg/kg	Oral	14 weeks	[125]
SIRT1	T cells	5 mM			[126]
mTORC1/AKT	Fibroblasts	10 mM			[127]
	Mice	200 mg/kg	Oral	17 days	[128]
	Cells	4 mM			[129]

### Metformin exerts its effect in an AMPK-independent manner

#### Metformin exerts its effects by restoring redox balance

**Fig. 3:**
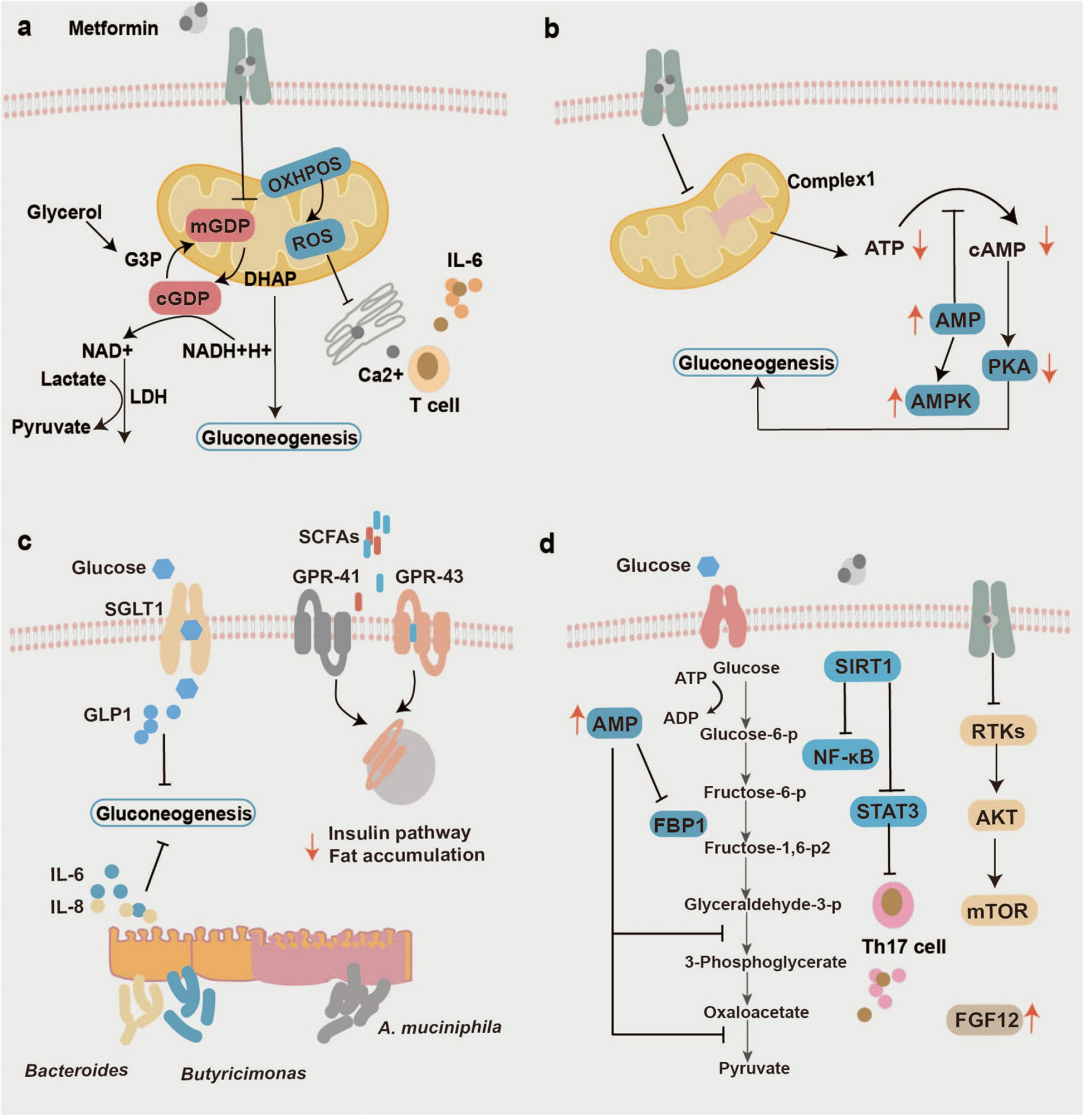
AMPK-independent mechanisms of action mediated by metformin treatment. **a** Metformin-induced restoration of redox balance. **b** Metformin-induced changes in mitochondria. **c** Metformin-induced modulation of gut microbiome. **d** metformin-induced regulation of signals, including FBP1, PP2A, FGF21, SIRT1 and mTOR

In addition to AMPK-dependent manner, it has also been reported that metformin elicits pleiotropic effects in an AMPK-independent way, such as restoring the cellular redox balance (Fig. 3a). Redox homeostasis is a balance between reactive oxygen species (ROS) and the antioxidant system, which is involved in diverse biological and pathological processes, such as metabolism, aging and cancer [130, 131]. Madiraju et al. uncovered that although chronic metformin treatment increased the phosphorylation of AMPK, acute metformin treatment does not lead to the activation of AMPK, it failed to increase the phosphorylation of ACC, a generally accepted signal for AMPK activation. The antihyperglycemic effect of metformin (50 mg/kg) is achieved by increasing the cytosolic redox state and decreasing the mitochondrial redox state, as determined by the ratio of NADH to NAD^+^, thus the G2PD activity and glycerophosphate dehydrogenase are inhibited, which results in blockade of lactate and glycerol entry into glucose, eventually, HGP is limited [13, 14]. Additionally, substantial evidence indicated that the remodeling redox status of metformin is relevant to different types of cancer. The apoptosis of acute myeloid leukemia (AML) cells is observed after treatment with metformin, which is mediated by reducing ROS via downregulation of oxidative phosphorylation (OXPHOS) [100]. The proliferation of pancreatic cells and osteosarcoma is also inhibited by metformin-mediated ROS downregulation [101, 102]. Moreover, metformin also enhances the sensitivity of esophageal squamous cell carcinoma and colorectal cancer to cisplatin in a redox-dependent way [103, 104]. Of note, the concentrations of metformin used in these cancer researches ranged from 1 to 10 mM, and they were all much higher than the clinically relevant metformin dose.

Besides, the significance of redox homeostasis on aging may offer an explanation for the metformin’s role in aging. Studies with erythrocytes confirmed the hypothesis that metformin could maintain redox homeostasis by reducing aging-related oxidative stress and strengthening antioxidant machinery to improve heme function [105–107]. In healthy older people, the ROS in CD4^+^ T cells could produce a Th17 inflammation profile, however, Bharath and his colleagues indicated that by increasing autophagy and improving mitochondrial function, the metformin(100 μM)-restored redox balance is able to alleviate this inflammation profile [42]. Furthermore, some severity and fatality cases of COVID-19, a currently pandemic disease, are likely relevant to elevated IL-6 levels [132], Previous studies revealed that the reduction of ROS by metformin(1 mM for cells and 150 mg/kg for mice models) is capable of inhibiting calcium release-activated channels(CRAC)-mediated Ca^2+^ release from the endoplasmic reticulum, consecutively, inhibiting interleukin 6(IL-6) release [108, 133]. Based on these results, the impediment of ROS/Ca^2+^/IL-6 pathway may be another explanation for the beneficial role of metformin in COVID-19 (Table 1).

#### Metformin exerts its effects in a mitochondria-dependent way

Since it was mentioned in 1950, it has been generally accepted that metformin has an inhibitory effect on mitochondrial biological function (Fig. 3b), based on convincing data from various cellular models, including rat, mouse and human hepatocytes [134]. The major function of mitochondria is producing ATP through oxidative phosphorylation, which is mediated by respiratory chain complex I. It has been reported that as a noncompetitive inhibitor, metformin enables binding to the Cys39-containing matrix loop of the mitochondrial complex I subunit ND3, however, data from bovine heart mitochondria indicated that the metformin is only a weaker inhibitor of complex I with an IC50 value of 19.4 ± 1.4 mM [135].

As the gluconeogenesis is highly dependent on energy production, consuming six ATP molecules per one glucose molecule synthesized, the metformin-mediated inhibition of mitochondrial biological function, which further results in a decrease in cellular ATP production, which may be another mechanism for its role in HGP reduction. With an AMPKα1/2 knockout mice model, Foretz et al. found that high-dose (≥ 250 μM) metformin treatment still inhibits HGP by decreasing ATP and increasing AMP [109]. In addition, by suppressing the mitochondrial electron transport chain, metformin upregulates the ratio of AMP to ATP, and the increased AMP subsequently inhibits adenylate cyclase to abrogate cAMP production, which further lowers PKA and fructose-2,6-bisphosphate1. These metformin-induced events eventually lead to a decrease in HGP. They further confirmed that in the AMPK-deficient mice and hepatocytes, metformin is still able to block the cAMP accumulation [110]. Apart from its effect on glucose metabolism, the effect of metformin on mitochondria is also plays a role in lipid metabolism. By increasing the biogenesis of mitochondria in brown adipose tissue, a tissue with a vast number of mitochondria, metformin (250 mg/kg) suppresses fatty acid uptake and promotes thermogenesis, exerting anti-obesity effects [111].

Besides its role in energy metabolism, metformin also affects cancer by regulating mitochondrial biogenesis. Dan et al. found that AMPK signaling changes could not fully explain the anticancer effect of metformin, and NAD + /NADH homeostasis and aspartate are also involved. As NAD + /NADH homeostasis and aspartate biosynthesis were previously reported to be critical for cancer cell proliferation, they indicated that metformin (1 mM for cells and 500 mg/kg for mice models) could suppress the proliferation of cancer cells by inducing the loss of NAD + /NADH homeostasis and downregulating aspartate biosynthesis levels through inhibiting mitochondrial complex I, which is also called NADH dehydrogenase [20, 112]. Moreover, the suppression of mitochondrial complex I by metformin (500 μM) also results in the enhanced glycolysis and a reduced citric acid cycle; subsequently, the cancer cells become energetically inefficient and their proliferation is inhibited [113].

Accordingly, mitochondria also participate in many other protective actions of metformin. In AMPK-deficient mice, the metformin treatment could still reduce infract size following ischemia reperfusion in an AKT-dependent way. This cardioprotective effect of metformin (0.05–2 mM) is executed by inhibiting mitochondrial complex I, consecutively, suppressing the attenuation of mitochondrial permeability pore (mPTP) opening [114, 115]. Besides, by utilizing a PD worm model established by knocking down bcat-1, a recent research reported that PD-like features are closely correlated with “mitochondrial hyperactivity”, and metformin(50 μM) could improve neuronal activity and motor function by reducing this “mitochondrial hyperactivity” [116]. Furthermore, it is well known that mitochondrial function can be affected by its morphology and that metformin can affect the morphology of mitochondria [136]. Increasing evidence suggests that mitochondrial abnormalities might be a key contributor to the generation of the Down syndrome(DS) phenotype, and some chromosome 21(Has21) genes also affect mitochondrial function and morphology [137–140]. Lucio et al. pointed out that metformin(500 μM) corrects the mitochondrial dysfunctions of human fibroblasts from DS foeti by restoring the mitochondria to a branched and elongated tubular morphology in a PGC-1-dependent way, thus, metformin presented a promising role in improving DS-associated pathologies [117].

However, the inhibition of mitochondrial complex I or G3P dehydrogenase by metformin blocks pyruvate carboxylase and promotes glycolysis, resulting in an increase in lactate production and a decrease in lactate metabolism. Therefore, if the patient has chronic kidney disease, which may impair the metformin excretion, and circulatory dysfunction and chronic liver disease, which may impair lactate clearance, metformin treatment increases the risk of lactic acidosis, a low-incidence but serious adverse effect of metformin [141, 142] (Table 1).

#### *Metformin exerts its effects *via* the modulation of gut microbiome*

Notably, the bioavailability of metformin in the gut is 300 times higher than that in the plasma. Accumulating evidence from preclinical studies has uncovered the role of metformin in gut microbiome modulation, including increasing the proportion of parts of the microbiome, such as A. muciniphila, Bacteroides, Butyricimonas, Megasphaera, and Prevotella, and decreasing the proportion of parts of the microbiome such as Anaerotruncus, Lactococcus, and Parabacteroides [143, 144]. Indeed, substantial data have demonstrated that gut microbiome dysbiosis puts contribution to various diseases, such as glucose metabolism, cancer, aging, and even acquired immunodeficiency syndrome(AIDS) [145–148], and increasing evidence indicated that the modulatory action of metformin on the gut microbiome is another mechanism that accounting for the pleiotropic effects of this drug (Fig. 3c).

According to previous studies, there is four gut microbiome-involved mechanisms exerting metformin’s glucose-lowering effects: (1) Increasing glucagon-like peptide-1(GLP-1) release. Pretreatment with *m*etformin (200 mg/kg) recoveried the *Lactobacillu* abundance in the upper intestine and prompted sodium-glucose cotransporter-1 (SGLT1) sensing, a critical negative signal for glucose absorption in the upper intestine, which caused the increased GLP-1 secretion, eventually lowering HGP [24, 149–152]. (2) Promotion of short-chain fatty acids (SCFAs) production. SCFAs, including butyrate, acetate, propionate and lactate, execute protective roles against insulin resistance by multiple pathways, such as G protein-coupled receptors -41(GPR-41) and GPR-43 [153, 154]. Metformin (200 mg/kg) could upregulate the levels of SCFAs by increasing the abundance of the SCFA-producing gut microbiome, such as *Butyricimonas spp, Allobaculum* [118, 155, 156]. (3) Reducing gut permeability. The association of glucose metabolism and gut permeability has been clarified in several studies, and increased gut permeability results in insulin resistance through increasing the lipopolysaccharide (LPS) levels and causing chronic inflammation [157, 158]. According to previous data, metformin is capable of increasing the proportion of *A. muciniphila* in the gut which could reduce the gut permeability by stimulating mucin production or tight-junction protein expression [119, 159–161]. (4) Modulating inflammation. As the close relationships between glucose metabolism and inflammation have been studied, a hypothesis attracted researchers’ interest that metformin might elicit glucose-lowering effect through modulating inflammation in a gut microbiome-dependent way. Lee and his colleagues have shown that metformin treatment (250 mg/kg) increases abundance of *Bacteroides and Butyricimonas* in the gut, and then inhibits IL-6 levels or IL-1β expression, which are negative contributors to lowering the glucose process [25].

In terms of the effect on modulating gut microbiome, the most studied cancer type influenced by metformin is colorectal cancer [162]. In summary, the underlying mechanisms, which is mainly regulated by gut microbiome or its catabolite and metabolite, could be divided into four categories: (1) suppressing inflammation through Toll-like receptors 1(TLR1)/TLR4 pathway, or pro-inflammatory cytokinc, such as IL-6, IL-17a, IL-18 [163]; (2) increasing anticarcinogenic metabolites, such as SCFAs, or decreasing carcinogenic metabolites, such as hydrogen sulphide [164]; (3) inhibiting genotoxins production, such as *B.frigilis* toxin, CDT [24, 165]; (4) regulating not only innate immune by P38 map kinase-1 (PMK-1)/P38, receptor for advanced glycation endproducts (RAGE) ligands pathway and cytokines, such as interferon–γ (IFN-γ) of natural killer (NK) cells, IL-12 of dendritic cells [166–168], but also adaptive immune by T cells infiltration [169, 170]. Of note, the metformin doses used in these studies are all much higher. Besides, with the Lox-Stop-Lox Kras G12D/ + mice model, Eibl et al. confirmed that metformin ( approximately 200 mg/kg) is able to reduce the incidence of pancreatic ductal adenocarcinoma by changing the duodenal microbiome in the mice models treated with high-fat diet [120]. Furthermore, as there has been convincing evidence that the immune checkpoint inhibitors (ICI) therapy and metformin exposure both increase the abundance of *A. muciniphila and Bifidobacterium spp* in the mice models and humans [171, 172], it is plausible to speculate that metformin-mediated modulation of the gut microbiome is capable of improving effectiveness of immunotherapy on cancers, which has been confirmed by a large number of prospective and retrospective studies [173–176].

Accordingly, the gut microbiome is closely associated with human life span, and gut microbiome dysbiosis plays an important role in aging development via affecting multiple processes [177–179]. For instance, the data from studying the African killifish model showed that the natural gut microbiome from young individuals has a life-extended impact on vertebrate models through inducing long-lasting systemic advantages. Hematopoietic development and terminal myeloid differentiation are also regulated by microbiome-inducible inflammation. Lucas et al. found that the percentage of resident T cells in the secondary lymphoid organ, which increases with age, is affected by gut microbiome [121, 180, 181]. As mentioned above, metformin has a profound influence on gut microbial composition and metabolism, taken together, it is plausible that the action of metformin in improving aging-related pathology and extending life span is relevant to its modulatory action on gut microbiome. This hypothesis is also consistent with the research conducted by Cabreiro and his colleagues with the C.elegans models, who presented that metformin (50 mM) can specifically prolong the life span of C.elegans by inhibiting the microbial folate cycle and reducing methionine [121]. Besides, as the increased gut permeability is also linked with inflammation in older adults, a risk factor for aging-related morbidities and mortalities, Yadav et al. found the metformin-regulated (100 mg/kg) gut microbiome has a protective role in aging by decreasing gut permeability and inflammation [119] (Table 1).

Noteworthy, studies have found a role of the gut microbiome modulated by metformin in inhibiting human immunodeficiency virus (HIV)-related inflammation [182]. The underlying mechanisms involved in the activation of tryptophan pathway are mediated by influencing tryptophan-catabolizing bacteria, and the improvement of the gut epithelial barrier mediated by *Akkermansia muciniphila* or other SCFA producing bacteria [183, 184].

#### Metformin exerts its effects by regulating several other signals

In addition to above mentioned mechanisms of action, studies have reported several other signaling which also affected by metformin, including FB1, PP2A, FGF21, SIRT1 and mTOR (Fig. 3d). Generally accepted as a rate-controlling enzyme in gluconeogenesis, fructose-1,6-bisphosphatase 1(FBP1) is able to catalyze the irreversible hydrolysis of fructose-1,6-bisphosphate (F-1,6-P2) to fructose- 6-phosphate (F6P), which can be inhibited by AMP and F-2,6-P2. In a mice model with a point mutation in FBP1, Roger and his colleagues uncovered that the glucose-lowering effect of metformin (250 mg/kg) is blunted, even though it still leads to the activation of AMPK. They concluded that FBP1 is a key regulator for the HGP inhibition of metformin, but does not depend on AMPK activation [122].

As high-glucose simulates cardiomyocyte apoptosis, metformin(500 μM) can exert its cardioprotective role by activating PP2A, thus reducing ROS production and inhibiting the proinflammatory response [123]. Furthermore, with the intermittent fasting model, Minucci and his colleagues found that the combination of hypoglycemia and metformin (200 mg/kg) could inhibit tumor growth by activating PP2A, a tumor suppressor, in the absence of AMPK. Mechanistically, metformin activates the PP2A-GSK3β-MCL-1 pathway by inhibiting cancerous Inhibitor Of PP2A (CIP2A), a PP2A inhibitor, and hypoglycemia upregulates the B56δ, a PP2A regulatory subunit, eventually, the active PP2A-B56δ has higher affinity for GSK3β [124].

It has been well accepted that fibroblast growth factor 21 (FGF21) is a critical regulator of glucose and lipid metabolism. Consistent with its function, some studies have found that the anti-obesity effects and glucose-lowering effects of metformin are also exerted by FGF21. Metformin suppresses adipocyte differentiation by increasing FGF21 expression in the liver and white adipocytes in an AMPK-independent way, thus eliciting its therapeutic effect on T2DM, obesity and fatty liver [125, 185].

Sirtuin 1 (SIRT1), an NAD + -dependent deacetylase, leads to an anti-inflammatory effect by suppressing NF-κB signaling through deacetylation of its p65 subunit. Song et al. showed that the SIRT1 can be activated in an AMPK-dependent manner [186]. It has been shown that metformin ameliorates inflammation of circulating peripheral blood mononuclear cells in patients with carotid artery atherosclerosis by inducing SIRT1 [187, 188]. In addition, the anticancer effect of metformin may also be relevant to SIRT1, as the metformin-induced SIRT1 is able to reduce the Th17 population and IL-17 levels in tumors by deacetylating STAT3 [126].

By utilizing an AMPKα1/α2 double-knockout MEF model, Kalender et al. discovered that the inhibition of mTORC1 by metformin is independent of AMPK, but in a Rag GTPase-dependent manner [127]. Additionally, in the AMPK-deficient MEFs, it was reported that metformin inhibited phosphorylation of RTKs and AKT/mTOR way [128]. Metformin suppresses the proliferation of the AMPK-deleted glioma by activating mTOR signaling [189]. Besides, metformin is reported to reduce the anticancer efficiency of cisplatin in an AKT-dependent manner, but not an AMPK-dependent manner, as metformin failed to further increase cisplatin-induced AMPK activation [129] (Table1).

## Clinical study

**Fig. 4:**
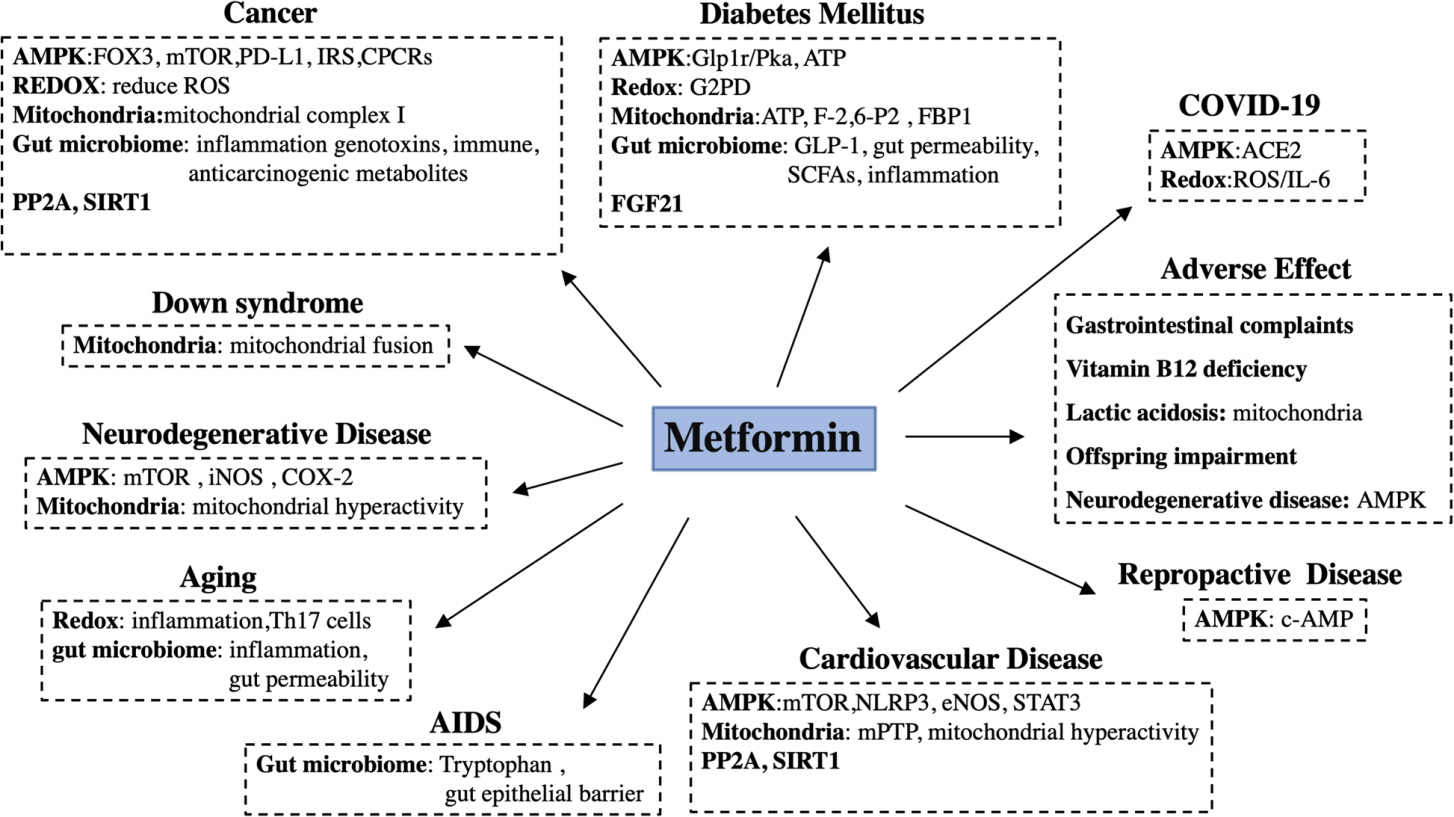
Summary of metformin in different diseases and the major related mechanism. Based on its mechanisms, metformin has potential beneficial for diabetes mellitus, cancer, neurodegenerative disease, aging, cardiovascular disease, reproductive disease, COVID-19, and even Down syndrome and AIDS, however, it is also companied with some adverse effect, including gastrointestinal complaints, vitamin B12 deficiency, lactic acidosis, offspring impairment and neurodegenerative disease

Based on the various of underlying mechanisms, through which metformin can affect some diseases, including diabetes mellitus, cardiovascular diseases, neurodegenerative diseases, reproductive disease, aging, cancer and COVID-19, researches considered that metfomin is possible to have therapeutic potential for these disease in the chilic, so they further conducted serious clinical studies and analyzed the outcomes of these diseases when treatment with or without metformin to explore the possibility for repurposing this old drug (Fig. 4).

### Clinical efficacy of metformin

#### Diabetes mellitus

Metformin monotherapy effectively improves blood glucose control and lipid concentration in patients with T2DM and was approved in the USA in 1994 [190]. Since then, metformin has been widely used as a first-line oral glucose-lowering medication for the management of T2DM in the clinic [2]. Accumulating studies and clinical trials demonstrate that metformin-based regimens are effective in the curation of T2DM [191]. Recent evidence indicated that the regimen of metformin in combination with other antihyperglycemic drugs, including troglitazone, dipeptidyl peptidase 4 (DPP4) inhibitors, glibenclamide, insulin, glucagon-like peptide 1 (GLP1) receptor agonists, and sodium-dependent glucose transporters 2 (SGLT2) inhibitors, presents a better therapeutic effect on controlling plasma glucose levels than metformin alone. So, compared to using metformin alone, the combined use of metformin and glibenclamide exhibited a better glucose-lowering effect [192]. For instance, in a 16-week, randomized,double-blind study, the data showed that the fasting plasma glucose(FPG) is signicficantly lower in the metformin/ glibenclamide group (9.4 mmol/l, 2.5 mg/500 mg) than in metformin alone group (13.0 mmol/l, 500 mg [193]. Similarly, the combination of metformin with troglitazone reduced the production of endogenous glucose and promoted the metabolism of peripheral glucose, consequently, presenting better control of the plasma glucose level in T2DM patients [194]. Moreover, adding metformin to insulin therapy was reported to have better therapeutic efficiency for T2DM patients [195]. Although the efficiency of the combination strategy on glucose-lowering is evident by these studies, more studies, especially on the side effects, should be conducted (Table 2).

**Table 2:** Summary of clinical studies of metformin

Diseases	Type of studies	Status	Characteristics	Year	Outcomes	Effect size	P-values / 95% CI	References
Diabetes mellitus	Randomized-controlled trial	Finished	29 patients to receive either metformin or troglitazone for three months	1998	Plasma glucose concentrations	Metformin, decreased by 20%; Troglitazone, decreased by 25%	*P* < 0.001	[192]
	Randomized controlled double-blind trial	Finished	390 patients to receive either the placebo or metformin, in addition to insulin therapy	2002	Mean daily glucose at 16 weeks	7.8 vs. 8.8 mmol/l	*P* = 0.006	[193]
Cardiovascular diseases	Randomized controlled trial	Finished	1,673 patients to receive either conventional or metformin	1998	Risk of myocardial infarction	39% reduction	*P* = 0.01	[196]
	Randomized controlled trial	Finished	4209 patients to receive either conventional therapy (dietary restriction) or intensive therapy (either sulfonylurea or insulin or, in overweight patients, metformin) for glucose control, 10-year follow-up	2008	Risk of myocardial infarction	33% reduction	*P* = 0.005	[197]
	Randomized controlled trial	Finished	304 patients to receive either glipizide (30 mg daily) or metformin (1.5 g daily) for 3 years	2013	Adjusted hazard ratio (HR) of cardiovascular events	0.54	*P* = 0.026	[198]
	Randomized controlled trial	Finished	36 HFrEF patients (ejection fraction 37 ± 8%; median age 66 years) were randomised to metformin (*n* = 19) or placebo (*n* = 17) for 3 months in addition to standard heart failure therapy	2020	Reduce myocardial oxygen uptake	17%	*P* = 0.01	[199]
	Randomized controlled trial	Ongoing	1,500 patients with T2DM and heart failure to receive either metformin (2000 mg / d) or placebo, and the follow-up period was expected to be 4 years	2021				[200]
Neurodegenerative diseases	Prospective, observational	Finished	1,037 community-dwelling older participants, 123 had diabetes; 67 received metformin	2020	OR of rate of cognitive decline	5.29	*P* = 0.05	[201]
	Retrospective	Finished	a representative cohort of 800,000 was obtained between 1996–01-01 and 2007–12-31	2012	HR	0.95	95% CI (0.53–1.71)	[202]
Reproductive diseases	Randomized controlled trial	Finished	487 patients to receive metformin (*n* = 244) or placebo (*n* = 243)	2019	OR of rate of miscarriage and preterm birth	0.50	*P* = 0.08	[203]
	Randomized controlled trial	Finished	153 patients to receive metformin (*n* = 77) or placebo (*n* = 76)	2016	incidence of moderate-severe OHSS	Placebo = 12.2%, metformin = 16%	*P* = 0.66	[204]
	Randomized controlled trial	Finished	357 obese pregnant women to receive either metformin or placebo	2020	OR of the rate of preeclampsia	0.17	95%CI (0.10–0.41)	[205]
	Randomized controlled trial	Finished	180 women with preterm pre-eclampsia between 26 + 0 to 31 + 6 weeks' gestation undergoing expectant management: 90 were randomized to extended-release metformin and 90 to placebo	2021	Median prolongation of gestation	17.5 days in the metformin group compared with 7.9 days in the placebo group	P = 0.057	[206]
Cancer	Delayed start randomized trial	Finished	100 patients were enrolled (51 in chemotherapy alone vs. 49 in metformin with chemotherapy arm)	2019	dose-limiting toxicities	6.1% in the metformin with chemotherapy arm compared to those who received only chemotherapy 7.8%	95% CI (0.39–0.92)	[207]
	Double blind phase II randomized trial	Finished	40 patients were randomized to receive metformin 850 mg po bid or placebo bid	2019	HR of PFS	1.2	95% CI (0.63–2.31)	[208]
	Phase II randomized trial	Finished	128 patients to receive metformin 250 mg orally per day or placebo tablets of identical appearance in the same regimen for a convenient duration of 7 to 14 days before surgery	2020	Infiltration of CD8^+^ T cells	-	*P* < 0.001	[209]
	RCT	Finished	40 patients with solid tumors who received metformin for concomitant diabetes and nivolumab as anticancer therapy	2022	PFS	?	*P* = 0.021	[210]
COVID-19	Retrospective	Finished	2,449 patients were enrolled, 1496 were to receive metformin and 953 were not	2020	Tracheal intubation and/or death within 7 days of admission	28.0% of metformin users (vs 29.0% in non-users)	*P* = 0.613	[211]
	Retrospective	Finished	422,554 patients who tested positive for SARS-CoV-2 were enrolled (23,327 metformin users; 8,639 metformin non-users)	2022	OR of the risk of total mortality	0.70	95% CI (0.66–0.75)	[212]
	Retrospective	Finished	24,722 subjects who tested negative for COVID-19 and 604 subjects who had a confirmed positive COVID-19 test were included	2021	OR of death for subjects	0.33	P = 0.0210	[213]
	RCT	Finished	663 patients receiving metformin and 660 patients not receiving metformin	2021	OR of death for subjects	0.58	95%CI, (0.35–0.94)	[214]
Adverse effects of metformin								
Vitamin B12 deficiency	Case–control study	Finished	155 patients with metformin-related vitamin B12 deficiency were compared with 310 matched controls	2006	OR for developing vitamin B12 deficiency	2.88	P < 0.001	[215]
	RCT	Finished	4,351 drug-naive individuals recently diagnosed with type 2 diabetes were assigned randomly to thiazolidinediones (TZDs), metformin, or sulfonylurea monotherapy and monitored for 5 years (1,343 metformin, 1,289 sulfonylurea, and 1,335 TZD users)	2020	OR for anemia	1.93	95% CI, (1.10–3.38)	[216]
	RCT	Finished	136 patients with Type 2 diabetes were divided into metformin exposed (*n* = 84) and non-metformin exposed groups (*n* = 52)	2013	Mean neuropathy score	5.72 ± 2.04 versus 4.62 ± 2.12	*P* = 0.0064	[217]
Lactic acidosis	Retrospective study	Finished	1,213 individuals with T2D, including 678 subjects (male, 53.8%) treated with metformin or metformin plus other anti-diabetic drugs (referred to as the metformin group) and 535 individuals (male, 49.9%) treated with anti-diabetic drugs other than metformin (referred to as the non-metformin group)	2020	Numbers of cases with severe COVID-19	32.60% versus 33.83%	*P* = 0.695	[218]
	Retrospective nested case–control study	Finished	29,264 patients with type 2 diabetes including 2,662 lactic acidosis cases and 26,602 matched controls	2020	adjusted hazard ratio of MALA	3.09	95%, CI (3.51–17.61)	[219]
Offspring impairment	RCT	Finished	257 pregnant women with PCOS participated with 274 pregnancies	2018	difference in height z score means	difference in means: 0.38 (0.07 to 0.69)	*P* = 0.017	[220]
	RCT	Finished	208 children assessed (28% of the original cohort). In Adelaide, 109 children (metformin *n* = 58, insulin *n* = 51) were assessed and, in Auckland, 99 (metformin *n* = 45, insulin *n* = 54) were assessed	2018	Infants with birth weight > 90th percentile	20.7% vs 5.9%	*P* = 0.029	[221]
	Population-based cohort study	Finished	1,996 children exposed to metformin during the fetal period and 1,932 treated with insulin	2019	Mean difference in the average weight z score	− 0.03	95% CI, (− 0.13 to 0.07)	[222]
		Finished	7,029 offspring were exposed to paternal diabetes medications, including insulins (*n* = 5,298), metformin (*n* = 1,451), and sulfonylureas (*n* = 647)		Percentage of genital birth defects	In metformin-exposed group vs. 0.24% in control group, aOR = 3.39	5%CI, (1.82- 6.30)	[49]
Neurodegenerative disease	Nested case–control study	Finished	Diabetes diagnosed ≥ 3 years before AD diagnosed (*n* = 7552) and controls received at least once metformin (*n* = 14,528)	2020	Adjusted odds ratios (aORs)	0.99	*P* = 0.775	[223]
	Case–control study	Finished	14,172 patients (7,086 AD and 7,086 matched controls)	2012	aOR of AD	1.71	95% CI, (1.12–2.60)	[224]
	Cohort study	Finished	4651 metformin users and an equal number of non-metformin users	2017	HR of PD	2.27	95% CI, (1.68–3.07)	[225]

#### Cardiovascular diseases

In 1988, the randomized UK Prospective Diabetes Study (UKPDS) trial with 1704 type 2 diabetes patients confirmed that metformin has cardiovascular protective effects. After a median follow-up of 10.7 years, this study confirmed that metformin treatment significantly reduces the risk of cardiovascular events in newly diagnosed T2DM patients compared to conventional therapy (diet control), and a 39% reduction in the risk of myocardial infarction was reported (*P* = 0.01) [196]. The follow-up for 10 years of the UKPDS intervention study found that the metformin has continuous cardiovascular benefits and reduces the risk of myocardial infarction by 33% (*P* = 0.005) [197]. Another randomized trial of 304 patients with T2DM demonstrated that there were a significantly fewer cardiovascular events for metformin than for sulfonylurea after 5 years of treatment [198]. A study by Larsen et.al showed that 3 months of metformin treatment (target dose of 1000 mg bid) in diabetic chronic heart failure patients with reduced ejection fraction (HFrEF) patients significantly reduced myocardial oxygen uptake by 17% (*P* = 0.01). Moreover, patients with higher plasma concentrations of metformin (> 1268 ng/ml) had better myocardial efficiency [199]. Recently, a large-scale clinical endpoint study of metformin for patients with chronic heart failure-Danish Heart Failure Study (DANHEART) is ongoing. This is a multicenter, randomized, double-blind, placebo-controlled study of 1500 patients with T2DM and heart failure. The dose of metformin was 2000 mg/d (1000 mg/d when eGFR was 35–60 ml/min/1.73 m^2^), and the follow-up period was expected to be 4 years. The primary endpoint is a composite of death, hospitalization for worsening heart failure, acute myocardial infarction or stroke. The results are expected to be published in 2023 [200]. Although, a large number of studies supported the improved clinical outcomes of patients with heart failure treated with metformin, especially HFpEF, it is not yet enough for the approval of metformin in treating heart failure, we believe its effect and mechanism deserve further exploration (Table 2).

#### Neurodegenerative diseases

Neurodegenerative diseases (ND) mainly include Alzheimer's disease (AD) and Parkinson's disease (PD). Accordingly, neurodegenerative diseases are characterized by misfolded and aggregated proteins in neurons, such as mutated α-conucleoprotein, tau protein, β-amyloid and Huntington's protein. These proteins are toxic to neurons because of their role in changing neuronal connectivity and plasticity and even activating of cell death signaling pathways. Besides, it is well documented that aging is a main risk factor for neurodegenerative diseases [226].

The effects of metformin on ND are controversial, and in this part, we focused on the beneficial effects. Currently, preclinical and clinical evidence mostly reveals that metformin seems to be a prime candidate for a clinical trial that aims to target AD. A meta-analysis described that metformin reduced the risk for developing AD in patients with T2DM [201]. Similarly, a prospective observational study found that metformin improves cognitive performance in elderly patients with diabetes [202]. Regarding PD, a retrospective study revealed that metformin can reduce the risk of PD in T2DM patients in a Taiwanese population [227]. Overall, current evidence mostly supports that metformin improvs cognitive performance and decreases the risk of AD, preventing AD (Table 2).

#### Reproductive disease

Worldwide, approximately 5–20% of reproductive-aged women worldwide are affected by PCOS worldwide, and most of them is characterized by hyperandrogenism, ovulatory dysfunction, insulin resistance and so on [228]. Researchers found that metformin treatment improved the pregnancy rate probabilities for women with PCOS [229, 230]. A randomized, double-blinded, placebo-controlled trial (PregMet2) showed a lower rate of miscarriage and preterm birth of women with PCOS treated with metformin (OR = 0.50, 95% CI, 0.22–1.08; *p* = 0.08), and no significant serious adverse events in either mothers or offspring were considered drug-related [203]. Also, several systemic studies have suggested that metformin improved assisted reproductive technique outcomes by lowering the rate of ovarian hyperstimulation syndrome (OHSS) during the treatment of PCOS [231, 232], however, a randomized placebo-controlled trial concluded that a short-term of metformin use did not reduce OHSS in a gonadotropin-releasing hormone antagonist cycle for patients with PCOS (*p* = 0.66) [204].

In addition, metformin can reduce the secretion of antiangiogenic factors from the placenta in a dose-dependent manner and mitigate endothelial dysfunction, thereby potentially promoting vasodilation in whole maternal omental blood vessels in patients with preeclampsia. A randomized controlled trial that included 357 obese pregnant women reported that using metformin during gestation can prevent preeclampsia (OR = 0.17, 95% CI 0.10–0.41) [205]. Another trial including 180 women with preterm preeclampsia showed that the median prolongation of gestation in the metformin group was 17.5 days compared with 7.9 days in the placebo group [206]. Despite its effects on the female reproductive system, metformin is also presented to improve semen parameters in obese males [233] (Table 2).

#### Aging

Although aging is an inevitable process for lives, researchers never stop exploring the mystery of aging and continue to devote themselves to extend lifespan. Given its anti-inflammation and restoration of redox balance effects, metformin was chosen to be investigated for its effect on the aging improvement. Some epidemiological studies have described that metformin can delay aging and reduce all-cause mortality in age-related diseases. Importantly, existing evidence has shown that metformin could extend life and health spans by acting as a geroprotective agent in diabetic patients and nondiabetic patients [234]. Besides, a randomized crossover trial has revealed that metformin affects both metabolic and nonmetabolic processes associated with aging in the 70-year-old participants [235]. Recently, an observational study demonstrated that metformin improves the overall survival of older diabetic patients compared with controls without diabetes [236]. It is also speculated that the glucose-lowering effects of metformin are a contributor to reduced risks of aging-related diseases, and thereby extending lifespan [237]. Of note, the randomized clinical trials, TAME (Targeting Aging with Metformin) and MILES (Metformin In Longevity Study), are investigating the potential of metformin as an anti-aging drug. To date, the results from MILES suggested that although metformin is possibly involved in antiaging transcriptional changes, its protective role in those subjects free of diseases remains controversial [238].The TAME trial proposed blood-based biomarkers of underlying biologic aging hallmarks: IL-6, TNFα-receptor I or II, C-reactive protein (CRP), growth differentiation factor15 (GDF15), insulin, IGF1, cystatin C, N-terminal pro brain natriuretic peptide (NT-proBNP), and hemoglobin A1c. Future trials to discover and validate future biomarkers were warranted [239] (Table 2).

#### Cancer

Based on the mechanisms of action, the clinical studies on the role of metformin on cancer, a fatal threat to humans, is continues increasing. Epidemiological studies have indicated that metformin can decrease the risk of developing cancer and reduce the cancer-related mortality. A series of clinical trials evaluating the anticancer effects of metformin in various solid cancers of different stages are ongoing. In these trials, metformin is used as a monotherapy, or in combination with chemotherapy, radiotherapy and immunotherapy. These trials mainly investigated the effects of metformin on survival outcomes, and the evalution marker includs overall survival (OS), progression free survival (PFS), and recurrence free survival, pathological response rate, and cancer proliferation markers. Also, limited trials have evaluated the maximum tolerance of metformin in specific tumors. Published data from these completed clinical trials showed promising results.

Several studies have estimated the role of metformin in the cancer prevention. Compared with the control group, the risk of adenomas was 0.60 (95% CI 0.39, 0.92) in individuals with a history of colorectal adenoma treated with a low dose of metformin (250 mg/day) for 1 year [240, 241]. While a similar population was given metformin at an escalating dose, from 500 to 2000 mg/day for 12 weeks, there was no significant difference in the primary endpoint and pS6K levels [242]. Metformin also displayed the consistent results in the prevention of endometrial cancer. Among patients with endometrioid endometrial cancer, administration of 850 mg/day metformin resulted in decreased cell proliferation (an 11.75% reduction in Ki-67, *P* = 0.008) [243, 244]. Besides, metabolic disorders, including obesity and T2DM, are related to the high risk and poor survival of pancreatic ductal adenocarcinoma (PDAC) [245, 246].Most epidemiologic studies have found that metformin treatment reduces the incidence of in PDAC patients with T2DM [247]. Taken together, the correlation between metformin treatment and cancer prevention indicates that cancer may benefit from metformin through the effect of metofmrin on the high risk factors of cancer (T2DM, obesity, etc.).

Besides, completed trials have demonstrated the role of metformin monotherapy before surgery [248]. In metformin-treated patients with breast cancer, Ki67 and homeostatic model assessment (HOMA)) were significantly reduced, and TdT-mediated dUTP nick end labeling (TUNEL) levels were also increased in the metformin-treated group [249]. Interestingly, TUNEL staining is higher in cancer patients without insulin resistance, while individuals who have insulin resistance show converse results [250]. Importantly, the estimation of the pAMPK change may depend on the cancer type. In breast cancer, pAMPK (*P* = 0.04) is significantly upregulated and pAKT is downregulated (*P* = 0.043). Ki67 and cleaved caspase-3 (*P* = 0.044) were obviously decreased compared with the control group [251]. Conversely, another phase-II trial found that pAMPK, pS6, pAKT, p-4E-BP-1 and ER expression were reduced after metformin treatment [243]. In prostate cancer, pAMPK showed no difference between the arm group and the experimental group with metformin monotherapy [252]. These biomarker changes revealed that metformin exerts anticancer effects in pleiotropic pathways.

Given the proposed preclinical data of metformin and cytotoxic reagents, the combination of chemotherapy and metformin has also been explored in clinical trials. Metformin in combination with established cytotoxic chemotherapy accounts for the majority of ongoing clinical trials of cancer treatment [253]. The results of in combination of metformin with anticancer agents are expected. As a adjuvant agent, metformin benefits the CSS (HR 0.58, CI 0.39–0.86) and OS (HR 0.69, CI 0.58–0.83) of patients with colorectal cancer. A meta-analysis has suggested that metformin, as a useful adjuvant agent, benefits the survival of patients with prostate cancer, particularly those after radical radiotherapy, however, in breast and urothelial cancer, no significant benefits were observed [254–256]. Meanwhile, in the adjuvant setting, a phase I study exhibited that metformin combined with chemotherapy had a lower rate of defined dose-limiting toxicities (DLTs) (6.1%) compared to those who received only chemotherapy (7.8%). AMPK phosphorylation increased by 4–6 folds, 46% showed stable disease and 28% of the patients who had quantifiable tumor markers showed favorable changes [207]. The other randomized, phase 2 clinical trial evaluated the efficacy of doxorubicin and cyclophosphamide versus chemotherapy alone plus metformin in nondiabetic patients with metastatic breast cancer. Moreover, it is found that insulin-resistant patients with HER2-negative metastatic breast cancer (HOMA ≥ 2.5) have significantly shorter PFS than those without insulin resistance. Metformin as a potential chemotherapeutic drug or effective adjuvant agent exerts an affordable, well-tolerated, and beneficial anticancer effects. However, another phase 2 trial showed that metformin showed no significant effect on RR, PFS or OS of chemotherapy plus metformin versus placebo in non-diabetic patients with metastatic breast cancer [208]. The inconsistent responses to adjuvant metformin therapy is attributed to the insulin status of patients with cancer. This suggests that the positive potential of metformin as a chemotherapeutic drug depends on the patients’ status and the simultaneous management of diabetes and cancer is necessary.

The impacts of metformin also vary by the tumor stage. Metformin decreased cancer-specific mortality rates and prolonged the survival of localized resectable PDAC patients with T2DM [257, 258].In contrast, a double-blind, randomized study of patients with advanced PDAC did not benefit from metformin when combined with gemcitabine and erlotinib [259]. Two meta-analyses described that metformin prolonged the survival of patients with local disease but not those with metastatic PDAC [260, 261]. Metformin had contradictory results in the survival outcome of cancer patients in the local and metastatic stages, indicating the importance of the cancer stage in the studies of metformin for cancer treatment.

Collectively, the existing studies showed inconsistent marker expression and survival outcomes with metformin use as an anticancer agent in different settings. Variation in study design and potential bias, especially time-dependent confounders affected by previous treatment, make it complex to explain the different results [262]. Besides, there is no enough evidence to analyze the impact of metformin dose and duration. Future randomized, controlled trials to elevate the dose and duration and the efficacy of metformin anticancer agents as are warranted.

Furthermore, considering the immunomodulatory properties of metformin, metformin has been combined with immunotherapy, in particular programmed death-1 (PD-1)/ PD-L1 immune checkpoint inhibitors [263]. A phase II clinical trial showed that low-dose metformin treatment (250 mg/day) to reprograms and activates the tumor immune microenvironment and may be a suitable immune response modifier for patients with esophageal squamous cell carcinoma [209]. An active tumor immune microenvironment is the foundation for checkpoint inhibitors to enhance the immune response. A study of 40 patients with solid tumors suggested that the combination of nivolumab and metformin is safe. Adverse events (AEs) occurred in 75% of patients (mainly fatigue, pruritus, rash, and asthenia). Grade 3 AEs occurred in only 20% of cases; no grade 4 AEs were observed. There is a statistically significant correlation between higher doses of metformin (> 1,000 mg daily) and longer PFS and OS [210]. Overall, low-dose metformin treatment is a tolerated and efficacious pretreatment/combination option to boost the effectiveness of checkpoint inhibitors. However, both patients with and without diabetes and tumors are heterogeneous.

Therefore, it might be rational to elevate the anticancer activity of metformin and survival outcomes according to the insulin resistance status and various stages of cancer of participants in future clinical trials. Further investigations on a possible synergistic effect of checkpoint inhibitors and metformin are recommended (Table 2).

#### COVID-19

Since it was first reported in 2019, the COVID-19 has spread throughout the world. According to data from World Health Organization COVID-19 dashboard on August 28, 2022, the cumulative number of cases is 596,873,121, including 6,459,684 deaths, so it is urgent to develop effective preventive and therapeutic methods. Apart from developing new drugs, researchers are also engaging in repurposing the old drugs to treat COVID-19. As discussed above, based on its effects on multiple pathogeneses, it is reasonable to speculate that metformin has therapeutic potential in COVID-19 treatment, and clinical data also support this hypothesis. Retrospective studies reported a significant metformin treatment-associated reduction in COVID-19 infection-related mortality in patients with T2DM [211–213]. A meta-analysis study, including 32 cohort studies with 2,916,231 diabetic COVID-19 patients, showed that metformin is significantly relevant to lower mortality with a pooled adjusted odds ratio (OR) of 0.78 (95% CI, 0.69–0.88) [264].

Moreover, clinical trials are also undergoing to reaffirm the beneficial effect of metformin on COVID-19 patients. Data from *ClinicalTrials.gov* [as of August 28, 2022; primary search keyword (condition/disease): COVID-19; secondary search keyword (other terms): metformin] exhibited only 3 clinical trials that are investigating the role of metformin in COVID-19 treatment. Among these, COVID-OUT, a phase 3, randomized, double-blind, placebo-controlled trial, has reported its result and showed that compared with the primary composite endpoint (hypoxemia, emergency department visit, hospitalization, or death) in nonhospitalized patients with COVID-19 between 663 patients receiving metformin and 660 patients not receiving metformin, the adjusted OR was 0.84 (95% CI, 0.66–1.09; *P* = 0.19), and there was no significant benefit for COVID-19-related primary events. However, through further analysis, it indicated that metformin has the potential to prevent the more severe components, including emergency department visits, hospitalization or death, as the adjusted OR was 0.58 (95% CI, 0.35–0.94) [214].

### Adverse effects of metformin

Besides efficacy and benefits, the safety of a drug needs to be fully considered. Due to its pleiotropic mechanism of action, metformin is not only beneficial to various diseases, conversely, it also results in several adverse effects, including gastrointestinal complaints, vitamin B12 deficiency, lactic acidosis, offspring impairment, and neurodegenerative diseases. When the patients are treated with metformin, clinicians need to closely monitor these adverse effects, especially those with fatal and irreversible harm. Generally, the most common adverse effect caused by metformin treatment is gastrointestinal complaints, which occurr in 2–63% of T2DM patients, and the complaints are diarrhea, nausea/vomiting, abdominal pain, flatulence, retching, dysgeusia. Although severe symptoms may lead to discontinuation in 5%-10% of metformin users [141, 265], the harm of gastrointestinal complaints is usually not fatal and irreversible, so we will mainly review the other adverse effects here (Table 2).

#### Vitamin B12 deficiency

Since it was first reported in 1969 [266], metformin-related vitamin B12 deficiency is prevalent in T2DM patients, the reported incidence varies from 5 to 40% [267–269]and decreased serum vitamin B12 levels vary from 14 to 30% [270, 271] in different studies. To date, although the mechanism by which metformin causes vitamin B12 deficiency is still unclear, clinical data have provided largely significant related-factors about the metformin-induced vitamin B12 deficiency. Ting et al. conducted a nested case–control study and found that the risk of vitamin B12 deficiency is dependent on the dose and duration of metformin use, for each 1 g/d metformin dose, the OR for developing vitamin B12 deficiency increased by 2.88 (95% CI, 2.15–3.87; *P* < 0.001). Compared with those receiving metformin for less than 3 years, among those using metformin for 3 years or more, the adjusted OR was 2.39 (95% CI, 1.46–3.91; *P* = 0.001) [215]. A hypothesis speculated that the mechanism responsible for metformin-mediated vitamin B12 deficiency is that metformin interferes with the calcium-dependent ileal membrane, which is responsible for the absorption of vitamin B12. Thus, Bauman et al. investigated the effect of calcium use on metformin-induced vitamin B12 deficiency, and the results confirmed that oral calcium supplementation reverses the decreased metformin-induced serum vitamin B12 level [272].

In addition, for the clinical manifestations of vitamin B12 deficiency mainly presenting as neurological and hematological symptoms, researchers further investigated the link between vitamin B12 deficiency and anemia or neuropathy. Regarding anemia, the RCTs, A Diabetes Outcome Progression Trial (ADOPT; *n* = 3,967) and UK Prospective Diabetes Study (UKPDS; *n* = 1,473), and an observational study, Genetics of Diabetes Audit and Research in Tayside Scotland (GoDARTS) population (*n* = 3,485), all give a similar result that the metformin-induced vitamin B12 deficiency is relevant to a higher risk of anemia [216]. Regarding to neuropathy, based on the severity of peripheral neuropathy (using the Toronto Clinical Scoring System (TCSS)) in both metformin users and non-metformin users, Singh et al. found an association of clinical neuropathy with metformin in T2DM patients (5.72 ± 2.04 in the metformin-exposed group versus 4.62 ± 2.12 in the metformin-unexposed group, *P* = 0.0064) [217]. Consequently, it is prudent to monitor vitamin B12 levels in metformin users, especially those with anemia or neuropathy manifestations. Because vitamin B12-caused neuropathy may be arrested with vitamin B12 or calcium supplementation, but diabetic neuropathy cannot (Table 2).

#### Lactic acidosis

According to clinical data, the lactate concentrations were 0.34 mmol/L higher in patients receiving metformin treatment [273], so metformin may increase the risk of lactic acidosis, especially in patients with kidney, liver and heart comorbidities. Interestingly, a recent retrospective study in a cohort of 1213 hospitalized diabetic COVID-19 patients displayed that metformin treatment is significantly relevant to a higher incidence of acidosis, especially in cases with severe COVID-19 complications [218]. Metformin-associated lactic acidosis (MALA), diagnosed by blood pH < 7.35, arterial lactate level > 5.0 mmol/L and metformin level > 5 mg/L [274], is an extremely rare event with an estimated incidence ≤ 10 events per 100,000 patients. However, its associated-mortality rates are up to 30–50% [46, 275], and a meta-analysis encompassing 177 patients with MALA from 44 studies showed an overall mortality of 36.2% (95% CI, 29.6–43.94%) with a median pH of 7.02 mmol/l and lactate of 14.45 mmol/l [276].

For this reason, a significant number of T2DM patients, who have a higher risk of MALA, were deprived of the benefits of metformin, but there is a debate in terms of the use of metformin in these patients.

Several studies have pointed out that the metformin would not increase the risk of MALA even in patients with eGFR 30–45 ml/min/1.73/m^2^, so FDA 2016 relaxed the renal restriction of metformin, recommending not to start using metformin if the eGFR is < 45 ml/min/1.73/m^2^ (CKD stage 3a) and not to continue using metformin if the eGFR is < 30 ml/min/1.73/m^2^ (CKD stage 3b) [277–279]. Over the years, new studies have reaffirmed the metformin safety in CKD patients with an eGFR ≥ 30 ml/min/1.73/m^2^. A community-based cohort study of 75,413 T2DM patients in the Geisinger health system showed that metformin treatment is only relevant to increased risk of MALA when the eGFR is < 30 ml/min/1.73/m^2^ (adjusted HR = 2.07, 95% CI, 1.33–3.22), and the results can be replicated by analyzing 67,578 new metformin users from 350 private US health systems [280]. A retrospective nested case‒control study in 2020 reported a consistent conclusion, by analyzing data from 320,882 diabetic CKD patients from the national VA Corporate Data Warehouse. Metformin exposure prior 3 months in patients with CKD stage 3a to 5 was associated with an elevated adjusted hazard of MALA (HR = 3.09, 95% CI 2.19–4.35 in CKD stage 3a; HR = 3.34, 95% CI 1.95–5.72 in CKD stage 3b; HR = 7.87, 95% CI 3.51–17.61 in CKD stage 4&5), but no association was evident in patients with CKD stage 1 or 2 (HR = 1.05, 95% CI 0.71–1.57) [219].

Although there have been a number of studies supporting the criteria of metformin use in CKD patients, further studies that test the precise criteria of tolerability and effectiveness of metformin in heart failure and chronic liver disease, even COVID-19, are still needed (Table 2).

### Offspring impairment

Studies considering the long-term effects of metformin use during or before pregnancy in offspring demonstrated conflicting results. Despite of the reported benefits, several studies also found that metformin may cause offspring impairment. Two randomized trials considered 4- to 9-year-old metformin-exposed children of mothers with gestational diabetes (GDM) or PCOS to acquire some long-term metabolic programming effects such as higher BMI and increased prevalence of overweight or obesity [220, 221].On the other hand, a study including 1,996 children exposed to metformin during the fetal period and 1,932 treated with insulin showed no differences in either child growth or neurodevelopment between both the groups [222]. Consequently, the role of metformin-exposure to pregnant women in offspring need to be further confirmed.

Although it has been reported that metformin is able to reduce serum testosterone levels [281], but in March 2022, Eisenberg and his colleagues proposed a surprising result regarding of the deleterious effect of metformin on offspring: preconception metformin treatment in fathers is associated with an elevated risk for major birth defects, particularly genital birth defects in boys. In this nationwide prospective registry-based cohort study, data from newborns and parents (1997–2016) through Denmark were collected, by analyzing sex and frequencies of major birth defects in offspring whose fathers used metformin during the development of fertilizing sperm. This research indicated that offspring exposed to metformin (*n* = 1451) had an increased birth defect frequency (aOR = 1.40, 95% CI, 1.08–1.82). For metformin-exposed offspring, genital birth defects were increased compared with the cohort (0.90% vs. 0.24%; aOR = 3.39, 95% CI, 1.82- 6.30), and more than 99% of genital birth defects occur in boys [49]. This is the first study to suggest that metformin use in fathers may be linked to birth defects; however, it is not sufficient to make any clinical changes to offer new medication advice for men with T2DM of reproductive age. More clinical studies are warranted to confirm these results, and further preclinical research is needed to explore the underlying mechanism of this phenomenon (Table 2).

#### Neurodegenerative disease

Although researches have shown a beneficial effect of metformin on ND, including AD and PD, the role of metformin in ND is still quite controversial, given some preclinical or clinical studies have reported that long-term metformin use may increase the risk of ND. In preclinical studies, when the C57BL/L mice received chronic metformin treatment, they exhibited impaired spatial memory and visual acuity, which indicated that metformin may have deleterious effects on the central nervous system [282]. Angela et al. further showed that metformin treatment causes enhanced gliosis in the ApoE-/- mice, a mice model of tauopathy that is usually used to study ND, and increases tau phosphorylation, resulting in elevated lipogenesis to aggravate the neurodegenerative process [283]. Moreover, some clinical studies further reaffirmed that metformin treatment is positively associated with ND. Regarding AD, scholars have reported that when the duration of metformin use is less than 3 years, the metformin may increase the risk of AD. A nested case‒control study of cases with diabetes diagnosed ≥ 3 years before AD diagnosis (*n* = 7552) and controls who received metformin (*n* = 14,528) at least once is conducted by Sluggett et al., the results showed that taking metformin 1–3 years increases AD risk [223]. Also, by studying data from 70,860 persons from the United Kingdom–based General Practice Research Database (GPRD), Imfeld et al. found that long-term users of metformin (≥ 60 prescriptions) had an increased risk of developing AD, compared to other antidiabetic drug users and nonusers (adjusted OR = 1.71, 95% CI, 1.12–2.60) [224]. Regarding PD, similar result was also observed. A cohort study by using Taiwan’s National Health Insurance Research Database to collect data from 4651 metformin users and an equal number of non-metformin users was performed by Kuan et al., through 12-year follow-up, and they pointed out that the metformin cohort presented a higher risk of PD than the non-metformin cohort (HR = 2.27, 95% CI, 1.68–3.07) [284]. Recently, a meta-analysis including 19 studies with 285,966 participants also supported that compared to non-metformin users or glitazone users, metformin monotherapy exhibits a significantly elevated risk of PD (OR = 1.66, 95% CI, 1.14–2.42) [34].

Furthermore, to explore the factors related to the adverse effect of metformin on ND, Moore et al. recruited participants from the Primary Research in Memory (PRIME) clinics study, the Australian Imaging, Biomarkers and Lifestyle (AIBL) study of aging and the Barwon region of southeastern Australia, they suggested that this phenomenon is possibly associated with another adverse effect of metformin, vitamin B12 deficiency. For this reason, a low serum vitamin B12 level (< 250 ρmol/L) is associated with ND, and taking vitamin B12 and calcium [225] which could promote vitamin B12 absorption supplements may alleviate metformin-associated ND outcomes [285]. Considering the controversial role of metformin in NDs, it is recommended to monitor the ND complications of T2DM patients when treated with metformin, and the serum vitamin B12 level could be a candidate biomarker (Table 2).

## Conclusion

After being used in T2DM treatment for more than 50 years, metformin, an old drug with magic effects, has attracted much interest in recent years because of its potential for repurposing. Metformin exerts various effects through pleiotropic mechanisms of action, including AMPK-dependent mechanism and AMPK-independent mechanism. Based on these diverse mechanisms, metformin has various influences on types of tissue including, but not limited, liver, gut, adipose, heart, vascular, brain, ovary, semen and even cancers.

The most involved mechanism of metformin’s action is AMPK activation. Emerging evidence has indicated that AMPK is not only a key effector of glucose and lipid metabolism, but involved in the regulation of various pathway, including: (1)Glp1r/Pka pathway and mitochondria, reducing HGP and improving insulin resistance; (2) mTOR/autophagy pathway, driving the cardiovascular protection and neuroprotection; (3)NLRP3, eNOS, STAT3, COX-2, iNOS or Smad3 pathways, mediating anti-inflammation; (4) FOX3, IRS, GCRP and PD-L1, executing anticancer; (5)cAMP pathway, improving reproductive system; (6)ACE2, probably protecting against COVID-19. Recently, researchers found when in the absence of AMPK, metformin is still able to exert its effect by restoring redox balance, affecting mitochondrial function, modulating gut microbiome and regulating several other signals. The investigation of these mechanisms has led us to further move toward understanding the role of metformin’s protective actions, for example, inhibiting HGP and cancers, anti-obesity, improving complications of COVID-19 and Down syndrome, cardioprotection and neuroprotection. It is worth mentioning that studies have pointed out by restoring the redox balance, metformin is able to improve aging via alleviating the related inflammation. What about other aspects of pharmacogenetics of metformin? The evidence summarized in this review suggests that due to its importance for human health, the gut microbiome is partly responsible for the beneficial effect of metformin. Through modulating the gut microbiome, which results in increased GLP-1 release and SCFAs production, reduced gut permeability, suppressed inflammation, decreased genotoxins production, and an improved immune system, metformin exerts glucose-lowering, anticancer, antiaging, and even anti-HIV effects. However, besides its beneficial roles, metformin also has some adverse effects, and the underlying mechanism is still unclear. Accordingly, the activation of AMPK may participate in the deleterious process of metformin, such as acute pancreatitis and AD. The promotion of glycolysis by metformin also increased the risk for lactic acidosis through increasing lactate production and decreasing lactate metabolism.

Under these circumstances, the roles of metformin in human homeostasis and disease are reaffirmed from a clinical standpoint. Consistent with the mechanism of metformin discussed above, the researches, whether observation studies or RCT studies, we reviewed here suggested that metformin has therapeutic potential for T2DM, cancer, cardiovascular disease, aging, COVID-19. However, clinical data also showed some adverse effects associated with metformin treatment, such as gastrointestinal complaints, vitamin B12 deficiency and lactic acidosis. Of note, the most interesting aspect of metformin is its controversial role in the reproductive system and nervous system. Regarding reproductive system, it is suggested that metformin is not only improves PCOS in reproductive aged women, but also increase the risk of birth defects via affecting the development of sperm. Regarding nervous system, clinical and preclinical evidences have confirmed the protective role of metformin in both AD and PD; on the other hand, some clinical studies have presented that metformin treatment may be a contributor to the cognitive impairment, and may be involved in the development of AD and PD. We considered that these different influences of metformin may be caused by the features of disease itself and the treatment duration of metformin.

However, despite the extensive preclinical and clinical data highlighting the potential therapeutic effect on an enormous spectrum of diseases, including T2DM, cardiovascular diseases, neurodegenerative diseases, reproductive disease, aging, cancer and COVID-19, for now, metformin is approved only for T2DM treatment in the clinic. We supposed that it is related to the metformin dose used in most researches, which is always much higher than its clinically relevant dose, and the debate about this huge dosage gap between plasma concentrations in the clinic and supraphysiological conditions is ongoing. According to the recommended dose, the chinically relevant dose of metformin is 50–100 μM for cells and 50–100 mg/kg for mice models. However, the doses of metformin used in most studies are more than 500 μM for in vitro researches or 250 mg/kg for in vivo researches (Table 1). Besides, the different dose of metformin also may lead to controversial effects; for example, in terms of mitochondria, a low-dose of metformin (75 μM) improves mitochondrial respiration through AMPK-mediated mitochondrial fission, in contrast, a high-dose of metformin (no less than 5 mM) inhibits mitochondrial respiration chain complex I. Therefore, the dose of metformin used in studies is critical for investigating its mechanism and it repurpose it in other indications.

Hence, to repurpose the metformin, in-depth mechanisms of action and more clinical evidence remain to be elucidated. Additionally, considering that in most diseases, only supra-pharmacologic doses of metformin work, which is possible to cause serious adverse effects and even toxicities, the application of metformin in other indications should be more prudent and further researches are needed to establish safer criteria for metformin use.

## Data Availability

Not applicable.
